# The Impact of Social Media on Disordered Eating: Insights from Israel

**DOI:** 10.3390/nu17010180

**Published:** 2025-01-03

**Authors:** Keren Dopelt, Nourit Houminer-Klepar

**Affiliations:** Department of Public Health, Ashkelon Academic College, Ashkelon 78211, Israel; nhouminer@gmail.com

**Keywords:** social media, disordered eating, BMI, nutrition, eating disorders, eating habits, diet, Instagram, Facebook, influencers, college students

## Abstract

Background: Social media platforms have become integral to daily life and increasingly disseminate health, nutrition, and food information. While these platforms can offer evidence-based nutrition education and meal planning guidance, a significant portion of content promotes unrealistic beauty standards and unhealthy weight-loss practices, potentially contributing to disordered eating behaviors. The increasing prevalence of disordered eating, characterized by abnormal eating behaviors and attitudes, has become a global public health concern. This study examines whether social media consumption correlates with disordered eating symptoms in Israeli college students. Methods: A cross-sectional study was conducted among college students at Ashkelon Academic College in Israel. The questionnaire assessed demographics, social media usage (platforms used, frequency, and content followed), body satisfaction, and disordered eating symptoms, measured via the Eating Attitudes Test (EAT-26). Results: The study sample comprised 580 students (73% were female and 27% were male) with a mean age of 27.87 years. Participants predominantly used multiple social media platforms, with a majority following food-, eating-, and health-related content. Despite having a normal BMI, a substantial number of participants reported body dissatisfaction. The results indicated a positive correlation between social media use and disordered eating symptoms. Individuals who frequently engaged with food-related content on social media exhibited higher levels of disordered eating. Moreover, body satisfaction emerged as a significant mediator in the relationship between social media use and disordered eating symptoms. Conclusions: The findings of this study support the growing body of evidence linking social media use to disordered eating behaviors among young adults. Developing targeted interventions that promote media literacy and foster body positivity is essential. Additionally, future research should explore the long-term effects of social media on eating behaviors and the effectiveness of different prevention strategies.

## 1. Introduction

Globally, there are approximately five billion users of social networks worldwide, with an average daily usage of 151 min per user [1]. Recent reports show that 91% of teenagers in the UK and US use social media, with over 50% checking their accounts at least once an hour [2]. Health, nutrition, and food have become common and prevalent topics on social networks [3,4]. Nutritional messages are disseminated by diverse sources, including accredited entities such as health organizations, doctors, and dietitians, as well as non-professional individuals like food bloggers, health and lifestyle “gurus”, celebrities, and influencers. The latter group often monetizes its content and competes for followers, compromising the quality and accuracy of information shared [5,6]. The proliferation of platforms and “influencers” on social media has created an online landscape that can be hazardous, marked by the rapid dissemination of large volumes of false information to millions of users [7]. Misinformation proliferates across fields, particularly in nutrition and dietary advice [8]. Misinformation on social media can influence attitudes, beliefs, norms, and behaviors, potentially compromising public health [9]. One notable consequence is the development of disordered eating [10].

Disordered eating is a complex pattern of abnormal eating behaviors and attitudes toward food, eating, and body image [11]. Although not a formal DSM-5-TR diagnosis, disordered eating is a significant risk factor for developing eating disorders and is associated with severe emotional and physical consequences, particularly when persistent [2,12,13]. Individuals with disordered eating exhibit excessive preoccupation with food and body image, manifesting as binge eating, restrictive dieting (such as fasting or skipping meals), purging, excessive exercise, emotional eating, and food-related anxiety. These behaviors can impair quality of life, generating feelings of guilt, shame, and loss of control [11,14].

Disordered eating predominantly affects adolescent girls and young women, often emerging during a critical developmental period [15]. Approximately 10% of adolescent girls and women engage in disordered eating behaviors, with about 3% meeting diagnostic criteria for an eating disorder [16]. Research consistently shows that up to 80% of young people in Western societies experience body dissatisfaction and engage in weight-loss behaviors, even at normal weight. Although these behaviors do not always indicate an eating disorder, they may cause significant distress and may heighten the risk of developing clinical eating disorders [17].

Israel exhibits the highest rates of disordered eating among adolescent girls, in comparison to 34 European countries, with approximately 50% currently dieting and 75% having dieted previously, despite only 15% being classified as overweight [18,19]. This trend is reflected in the increasing prevalence of eating-related pathology, with 20–22% of Israeli adolescent girls, particularly those aged 16–18, exhibiting symptoms of potential eating disorders [19]. Media messages promoting thinness exert greater influence on body image and disordered eating than family and friends in this population [18]. While disordered eating affects all genders and ages, women consistently demonstrate a higher prevalence, severity, and diversity of symptoms [15,17,20,21,22,23].

Eating disorders affect 8.4% of women, compared to 2.2% of men, according to a meta-analysis of 94 studies across Asia, Europe, and North America [2]. Though these conditions can emerge at any age, adolescence is a critical period for onset and often initiates a persistent pattern [16].Youth constitute a high-risk population [2] with 13% experiencing eating disorders, and 15–47% engaging in disordered eating behaviors [24]. College students show particular vulnerability, with nearly half reporting disordered eating behaviors [17]. Given the established link between negative body image and the development of eating disorders and disordered eating behaviors, these statistics underscore the urgent need for youth-focused prevention and intervention efforts.

### The Relationship Between Social Media Use and Disordered Eating

Social media use correlated with increased disordered eating, body image dissatisfaction, and negative mental health outcomes among young people [2,15,24,25,26]. The widespread promotion of unrealistic beauty standards on these platforms intensifies social comparison and contributes to body image concerns [27]. Repeated exposure to idealized body images exacerbates depression, low self-esteem, and preoccupation with weight and shape, particularly among young women [15,16,25].

Research consistently demonstrates that social media use is a significant risk factor for disordered eating among young people [16,28]. Social media algorithms often reinforce existing user preferences, creating information “bubbles” that limit exposure to diverse perspectives [29]. The messages conveyed through social media particularly appeal to young people, who are susceptible to social pressures and often lack critical media literacy [24]. A study conducted in Israel found a strong correlation between increased social media use and elevated levels of eating-related pathology, including body dissatisfaction, disordered eating behaviors, and drive for thinness, among adolescent girls [18].

A study examining the relationship between social media use and disordered eating behaviors among female students demonstrated a strong association between high levels of body dissatisfaction and disordered eating attitudes. Additionally, women reported that social media’s unrealistic and unattainable beauty ideals contributed to these negative body perceptions and promoted unhealthy weight management behaviors, such as dieting and disordered eating. Given the developmental stage of young women, characterized by physical changes and heightened body image concerns, the influence of social media on disordered eating remains particularly pronounced [16]. Wilksch et al. [30] reported disordered eating behaviors in 52% of girls and 45% of boys, with strict dieting and excessive exercise as common manifestations. The study found a positive association between frequent social media use, particularly on Instagram, and increased disordered eating behaviors, primarily among young women. These findings align with previous research indicating a correlation between increased social media engagement and increased risk for disordered eating behaviors [15,30].

Social media platforms expose users to a vast array of nutritional information, much of which may lack scientific credibility. This potential for misinformation, coupled with the platforms’ influence on body image and eating behaviors, may contribute to disordered eating patterns, which can impair nutritional status and health over time. Social media’s influence on dietary habits is therefore crucial. While research has examined the impact of social media on adolescent eating behaviors in Israel, the influence on the broader student population remains understudied, even though disordered eating is more common among young people. This study aims to examine the relationship between social media use and disordered eating symptoms among Israeli students. The research questions are as follows: Is increased social media consumption positively associated with lower body satisfaction among college students? Do individuals following food-related social media content show higher levels of disordered eating symptoms than non-followers? Are there significant differences in the prevalence of disordered eating symptoms between male and female college students? Does body satisfaction mediate the relationship between social media usage and disordered eating symptoms? We hypothesize that increased social media consumption will be positively associated with lower body satisfaction and higher levels of disordered eating symptoms. Additionally, we anticipate that individuals following food-related content will exhibit more disordered eating symptoms compared to those who do not. Furthermore, women are expected to exhibit more symptoms of disordered eating compared to men. Finally, we propose that body satisfaction mediates the relationship between social media use and disordered eating symptoms.

## 2. Materials and Methods

### 2.1. Procedure

We conducted a cross-sectional study. Ashkelon Academic College Ethics Committee approved the protocol (approval #46-2024). We programmed the survey questionnaire via Qualtrics (Qualtrics, Provo, UT, USA). On 28 January 2024, a link to the questionnaire was emailed to all students via the college’s computing infrastructure. A reminder was sent after two weeks, and on 25 February 2024, the survey was closed. The average response time was approximately 5 min. Of the 767 students who accessed the survey, 710 initiated it and 580 completed it, representing a 76% completion rate among those who started the survey and 14% of the total college student population. Students verified their informed consent and voluntary agreement to participate in the survey by completing the questionnaire. No question was mandatory, and students could halt their participation at any stage.

### 2.2. Tools

We collected data using a self-administered online questionnaire. Participants were assured of anonymity and provided with a clear study overview upon initiation. A pilot test was conducted with ten individuals unrelated to the study to assess questionnaire clarity. Based on feedback, three questions were revised. The questionnaire underwent content validation by three experts: a nutritionist, a psychologist, and a public health physician. The questionnaire consisted of three sections:

1. Demographic and general information: gender, age, marital status, religion, college department, height, weight, and body satisfaction (“Are you satisfied with the shape of your body?” on a scale of 1 (not at all) to 5 (strongly)).
2. Social media usage: Participants self-reported platform use (Instagram, Facebook, YouTube, TikTok, and Other), estimated daily usage, and engagement with food-, eating-, and health-related content (average time spent daily, types of accounts followed, how often they interact with these accounts, and diet changes affected by social media). For the purpose of the current study, platforms that are not primarily focused on disseminating nutritional information (WhatsApp, Telegram, Snapchat, Twitter, and similar platforms) were not included.
3. The Eating Attitudes Test (EAT-26) is widely used as a standardized self-reporting tool widely used to assess disordered eating symptoms [31]. The Israeli Ministry of Health’s National Psychological Unit translated the questionnaire into Hebrew. The EAT-26 is highly valued for screening purposes. It is applied across various contexts to evaluate the risk of disordered eating. The questionnaire comprises three components: diet, avoidance of fatty foods, and external appearance; bulimic symptoms and preoccupation with food; and self-control overeating habits. Items are measured on a Likert scale of 6 points (0 = never, 5 = always). Responses to questions are scored as follows: never/rarely/sometimes = 0, often = 1, usually = 2, and always = 3. Higher scores indicate more severe eating attitudes and behavioral issues. A total score of 20 or higher suggests a potential eating disorder. Evidence suggests that the EAT-26 scores are highly reliable (e.g., Cronbach’s alpha = 0.91 and Pearson r = 0.98) and valid (e.g., criterion validity = 0.90) in both general populations and patient samples [32,33,34]. In a recent study conducted among young women in Israel, the Cronbach’s alpha reliability for the overall test score was 0.86 [35]. The current study’s internal consistency was Cronbach’s α = 0.87.

### 2.3. Data Analysis

Data were analyzed using SPSS v.29 (IBM, Armonk, NY, USA). Independent samples t-tests examined group differences based on gender, marital status, having children, religion, and following food- and eating-related content. Differences between Body Mass Index (BMI) categories were examined using one-way analyses of variance (ANOVA) with Bonferroni correction. Pearson correlations assessed the relationships between age, body satisfaction, time spent on social media, and disordered eating. The PROCESS macro (Model 4) [36] was used to test the mediating role of body satisfaction in the relationship between social media use and disordered eating. A two-sided *p*-value < 0.05 was considered statistically significant.

## 3. Results

### 3.1. Sample Characteristics

A total of 580 students participated in this study. The sample comprised predominantly female participants (73%), with 51% being in relationships, 27% having children, and 81% Jewish. Most students were enrolled in the Faculty of Social Sciences (57%), followed by the Faculty of Health Sciences (26%), and Computer Science and Management (17%). The age range of participants was 18 to 60, with a mean of 27.87 ± 8.91 years. The survey population resembles the college population in terms of gender, age, and faculty composition. Regarding body image, 25% of participants reported dissatisfaction, 34% moderate satisfaction, and 41% satisfaction. Their BMI ranged from 15.2 to 47.1, with a mean of 24.5 ± 5.15. Over half of the participants fell within the normal BMI range (55%), 7% were underweight, 22% were overweight, and 16% were classified as obese. The sample characteristics are shown in Table 1.

### 3.2. Social Media Use

Social media platform use was assessed among participants. About 11% have no social media accounts at all (n = 64), 72% have Instagram (n = 415), 67% have Facebook (n = 386), 51% have YouTube (n = 295), 40% have TikTok (n = 242), and 4% (n = 24) mentioned “other” (Twitter). Platform subscription patterns showed 18% using one platform (n = 102), 22% two platforms (n = 124), 25% three platforms (n = 143), 23% four platforms (n = 135), and 1% five platforms.

Daily social media consumption was estimated among platform users (n = 516). Time spent ranged from less than one hour (17%, n = 89) to over three hours (30%, n = 155) per day. Two-thirds of social media users follow accounts focused on food, eating, and health (n = 344, 67%).

The interaction frequency varied among students following accounts focused on food, eating, and health (n = 344). A total of 15% engaged monthly (n = 53), 8% less than once a week (n = 26), 18% once a week (n = 63), 31% multiple times a week (n = 107), and 28% interacted with these accounts daily (n = 95).

Students following accounts focused on food, eating, and health (n = 344) reported following content related to the following: recipes and cooking tips (n = 273, 47%), physical activity and workout programs (n = 181, 31%), healthy lifestyle advice (n = 150, 26%), and weight loss/dieting information (n = 100, 17%).

### 3.3. Changes in Diet Due to Social Media Use

Among participants following food-, eating-, and health-related accounts (n = 344), a majority (87%, n = 299) reported making dietary modifications based on social media content. The most common changes included increased water consumption (50%), increased vegetable intake (39%), reduced sugar intake (29%), increased protein intake (27%), initiation of dietary supplements (22%), reduced calorie intake (23%), reduced carbohydrate intake (20%), reduced fat intake (17%), meal skipping or reduction (14%), and reduced gluten intake (6%).

### 3.4. Disordered Eating

The EAT-26 questionnaire responses were scored as follows: “never”, “rarely”, “sometimes” = 0, “often” = 1, “very often” = 2, and “always” = 3. The distribution of the EAT-26 questionnaire responses is presented in Table 2.

For the disordered eating variable, scoring was conducted following response categorization. A total score of 20 or above indicated pathological eating. In this sample, 12% of participants (n = 67) exhibited a pathological eating pattern.

### 3.5. Relationships Between Study Variables and Symptoms of Disordered Eating

Table 3 presents the relationships between study variables and symptoms of disordered eating.

We found that female students reported higher levels of disordered eating compared to males. Additionally, students without children reported greater disordered eating than students with children. Furthermore, students who followed content related to food and eating showed a decreasing trend in disordered eating symptoms.

Statistically significant negative correlations were observed between body satisfaction and disordered eating (r = −0.23, effect size = 0.05, *p* < 0.001), indicating that lower body satisfaction correlated with higher levels of disordered eating. Daily social media use exhibited significant positive correlations with disordered eating symptoms (r = 0.15, effect size = 0.02, *p* < 0.001), suggesting that increased social media use correlated with higher levels of disordered eating symptoms.

### 3.6. Body Satisfaction Mediates the Relationship Between Social Media Usage and Disordered Eating

**Figure 1:**
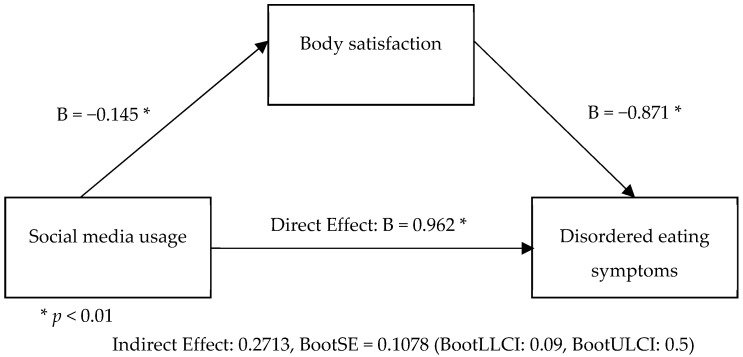
Body satisfaction mediates the relationship between social media usage and disordered eating.

A mediation analysis using the PROCESS macro (Model 4) was conducted to examine the indirect effect of social media usage on disordered eating through body satisfaction. Social media usage (X) was entered as the independent variable, body satisfaction (M) as the mediator, and disordered eating (Y) as the dependent variable. The model, as presented in Figure 1, showed that social media usage significantly predicted lower body satisfaction (B = −0.145, SE = 0.045, *p* = 0.0015), explaining 1.95% of the variance in body satisfaction (R^2^ = 0.0195, F(1, 514) = 10.23, *p* = 0.0015).

In turn, body satisfaction significantly predicted disordered eating (B = −1.871, SE = 0.333, *p* < 0.001), with lower body satisfaction associated with higher disordered eating scores. Social media usage also had a significant direct effect on disordered eating (B = 0.963, SE = 0.346, *p* = 0.0056), explaining 7.99% of the variance in disordered eating (R^2^ = 0.0799, F(2, 513) = 22.28, *p* < 0.001).

The indirect effect of social media usage on disordered eating via body satisfaction was statistically significant (Effect = 0.271, BootSE = 0.108, BootLLCI = 0.092, BootULCI = 0.513), as indicated by the bootstrapped 95% confidence intervals, based on 5000 bootstrap samples, which did not contain zero. These results suggest that body satisfaction partially mediates the relationship between social media usage and disordered eating, with social media usage contributing both directly and indirectly (via decreased body satisfaction) to increased disordered eating symptoms.

## 4. Discussion

Nutrition, a critical component of human health, has become a prominent topic on social media. However, the abundance of nutritional information online may negatively influence eating behaviors [37]. Our findings indicate that most participants reported spending over three hours daily on social media, with a quarter utilizing at least three platforms. Among social media users, 68% followed food-, eating-, or health-related accounts, and 28% interacted with this content daily. These findings align with previous research highlighting the pervasive influence of social media on daily life [2,38,39].

Social media exposure demonstrated a mixed impact on dietary behaviors. A majority of participants reported adopting healthier eating habits, including increased water and vegetable consumption and reduced sugar and caloric intake. Conversely, a subset (16%) described negative consequences, such as meal skipping. These findings underscore the dual nature of social media’s influence on dietary habits [40]. The widespread use of social media as a health information source is evident, with nearly 50% of US adults and 70% of the Chinese population relying on it for health management and education, respectively [9]. Qualitative studies support social media’s potential to promote food literacy, including tips on cooking, meal planning, and nutritional information, such as promoting fruits, vegetables, and whole grains [26,41].

Aparicio-Martinez et al. [16] noted that a third of women worldwide have experienced disordered eating at some point in their lives. Adolescence and young adulthood are particularly vulnerable periods for the onset of disordered eating, with prevalence estimates reaching 10% in the general population [16,17]. Findings from the current study align with these estimates, as 12% of participants reported disordered eating symptoms.

Additionally, it was found that individuals following food- and eating-related content exhibited higher levels of disordered eating compared to those who did not. Evidently, exposure to idealized body images and weight-loss narratives prevalent on social media may contribute to body dissatisfaction and disordered eating behaviors [42,43]. This process is particularly severe when messages are directed at young people [44]. A central aspect of social media under investigation by research is its role in promoting negative social comparison and its relationship with subjective well-being and unhealthy behaviors [45]. This connection is theoretically based on the idea that social comparison reduces happiness. Furthermore, findings suggest that social media users tend to engage in impression management practices. This means they may report fewer negative experiences or digitally alter the content they present, even limiting the posting of unflattering images on their online profiles. This manipulation of social media presentation to create a filtered image may increase the likelihood that published content reflects an idealized portrayal, facilitates appearance comparisons among users, and ultimately undermining their emotional well-being [45].

In our study, women reported significantly higher levels of disordered eating symptoms compared to men. Eating disorders typically occur in women in their twenties or during adolescence. Women aged 18–29 may be at particular risk for body dissatisfaction and disordered eating practices due to weight fluctuations and body changes commonly experienced during this life stage [16]. These findings align with previous research emphasizing the pronounced gender disparity in disordered eating [2,15,17,20,21,22,23,30,46,47,48].

This current study also found a negative association between body satisfaction and disordered eating, aligning with previous research indicating that body dissatisfaction is a significant predictor of disordered eating behaviors and can consequently lead to serious long-term health consequences [49]. Sanzari et al. [50] identified a link between exposure to thin ideal images on social media and reduced body image satisfaction, self-esteem, and increased body dissatisfaction among young women, as well as a relationship between media use and the development of problematic eating habits [51,52]. Israeli studies specifically highlight the association between prolonged social media use and heightened body image concerns, as well as disordered eating behaviors, including a drive for thinness, bulimia, and negative body image [18].

Body satisfaction emerged as a significant mediator in the relationship between social media use and disordered eating symptoms. The focus on self-objectification behaviors within social networks can act as a mediating factor in developing body dissatisfaction. The term “self-objectification” describes the behavior in which individuals regard themselves as objects to be evaluated by others [29]. In other words, they prioritize how others perceive them rather than how they feel about themselves. Long-term outcomes of self-objectification include body shame, excessive preoccupation with appearance, appearance anxiety, internalization of the thin ideal (emphasizing achieving a thin body), and an increased risk of disordered eating behaviors [29,49]. Murray and Goldfield [17] reported that among student populations, 49% exhibited disordered eating behaviors, with 80% of female students reporting body dissatisfaction. This figure is concerning, given that negative body image is a significant risk factor for the development of clinical eating disorders and disordered eating behaviors. Female students may be at heightened risk for body dissatisfaction and disordered eating due to the body changes and weight fluctuations commonly experienced during this life stage. Additionally, women who reported higher levels of body dissatisfaction and body-related concerns tended to spend more time on social media, which was associated with disordered eating behaviors [16].

The findings of this study can be contextualized within the frameworks of the sociocultural model of eating disorders [53] and self-objectification theory [54], both of which provide critical insights into the observed relationships between social media use, body dissatisfaction, and disordered eating symptoms. The sociocultural model highlights how societal beauty standards, perpetuated and amplified by social media platforms, promote the internalization of the thin ideal. This internalization drives individuals, particularly young women, to engage in appearance-focused behaviors and attitudes, often leading to body dissatisfaction and disordered eating. Furthermore, self-objectification theory explains how the image-centric nature of social media fosters a focus on external appearances, encouraging users to view their bodies from the perspective of others. This self-objectification is exacerbated by features such as likes and comments, which reinforce appearance-based social comparison. The mediation analysis in our study aligns with these theoretical perspectives, demonstrating how body dissatisfaction acts as a critical mechanism through which social media use contributes to disordered eating behaviors. By situating these findings within these frameworks, this study underscores the pressing need for interventions focusing on media literacy and body positivity to mitigate the adverse impacts of social media on young adults.

### Study Limitations

The current study was conducted exclusively among students from Ashkelon Academic College, which limits the generalizability of the findings to the broader student population across the country. Additionally, 72% of the sample consisted of women, potentially introducing bias into the results. However, this gender distribution reflects the proportion of women enrolled at the college. Moreover, we did not incorporate objective nutritional assessment tools in the survey; instead, we relied on the students’ self-evaluations of their dietary habits. Finally, we utilized a self-reported questionnaire to measure the variables, which necessitates caution in interpreting the results due to potential response biases. To strengthen the validity of our findings, future research should include follow-up studies that directly assess social media usage patterns and their potential impact on restrictive diets and the development of unhealthy eating habits.

While this study provides valuable insights into the relationship between social media use, body satisfaction, and disordered eating behaviors, it is important to acknowledge certain limitations regarding potential confounding factors. Variables such as mental health status, socioeconomic background, and prior diagnoses of eating disorders were not directly measured or controlled in this study. These factors are significant influencers of disordered eating behaviors and may interact with social media use, potentially exacerbating or mitigating these behaviors. For instance, individuals with preexisting mental health conditions, such as anxiety or depression, may be particularly vulnerable to the negative effects of social media on body image. Similarly, socioeconomic disparities could affect access to social media platforms, exposure to specific content, and the availability of resources to counteract harmful influences. Future research should aim to account for these variables to provide a more nuanced understanding of the complex interplay between social media use and disordered eating behaviors. Including these factors would improve the generalizability of findings and allow for a clearer delineation of potential causal pathways.

## 5. Conclusions

Social media platforms are powerful agents in shaping beauty standards, cultural values, and dietary norms. Research consistently highlights social media’s role in fostering disordered eating behaviors and negative body image among young people. This study contributes to a deeper understanding of the relationship between social media use and disordered eating behaviors, aligning with existing literature both domestically and internationally, across diverse population groups. However, the research also underscores the potential of social media to positively influence the acquisition of healthy eating habits when used effectively. Social media can serve as a valuable tool for disseminating professional knowledge, raising awareness, and educating users about health and nutritional literacy. It offers opportunities to share evidence-based information from qualified professionals and promote healthier lifestyles. To realize this potential, stronger regulations are needed for content shared on social media, particularly advertisements for products and services that may pose risks to public health. In light of these findings, we recommend implementing an intervention program focused on online health literacy and nutrition, led by the Faculty of Health Sciences. It is crucial to include a mandatory lecture on critical media literacy for all college students. Additionally, raising awareness about online nutrition literacy through targeted messages to students containing healthy eating tips is essential. Furthermore, establishing online support groups dedicated to nutrition literacy and creating social media pages where students can share their experiences and insights is highly recommended. Finally, the Ministry of Health should leverage various social media platforms to disseminate scientifically validated nutritional messages and guide the public in identifying self-proclaimed ‘experts’. An effective intervention strategy may involve establishing on-campus support groups and counseling services aimed at addressing body image concerns and promoting healthy eating habits. Collaborations with social media platforms can amplify body-positive messages and ensure the dissemination of scientifically accurate nutrition-related content. Advocacy efforts should prioritize implementing policies to regulate influencer content, ensuring transparency and accuracy in posts related to nutrition and health. Additionally, developing interactive applications can empower users by promoting healthy eating habits, fostering body positivity, and providing tools to identify and counter harmful content. Digital intervention programs, including virtual workshops, webinars, and curated resources, can further mitigate disordered eating behaviors exacerbated by social media influence. These interventions should be promoted through integrated mid-level cross-sectoral collaborations rather than isolated top-down approaches [55]. Social entrepreneurs can take part in such programs out of a desire to improve the existing situation and positively impact public health [56]. This integrated strategy can help reduce exposure to messages that may harm public health.

Further research is essential to elucidate the complex relationship between social media and eating behaviors, identifying factors that facilitate or hinder positive outcomes. A comparison with non-Israeli populations could provide valuable insights into cultural differences in the impact of social media. Research in diverse cultural and geographical settings may reveal whether similar patterns exist globally. Moreover, longitudinal studies should examine the long-term effects of social media use on eating behaviors and body satisfaction, helping to establish causality and identify trends over time. Furthermore, future research should explore the differential impact of specific social media platforms (e.g., Instagram vs. TikTok) on disordered eating symptoms and body image. By building upon existing knowledge and disseminating findings from the current literature, this study contributes to the growing body of research aimed at harnessing the potential of social media to improve public health through enhanced online health literacy and nutritional education. Future studies could also incorporate interviews with a sample of students to explore their experiences regarding the intersection of social media with nutrition and health.

## Figures and Tables

**Table 1:** The sample characteristics.

Characteristics	n	%
Gender:		
Male	159	27
Female	421	73
In relationship	297	51
Have children	155	27
Jewish	468	81
Faculty:		
Social Sciences	333	57
Health Sciences	150	26
Computers and Management	97	17
Body satisfaction:		
Weak (answers 1 + 2)	143	25
Moderate (answer 3)	196	34
Strong (answers 4 + 5)	241	41
BMI:		
Underweight (BMI < 18.5)	42	7
Normal (18–24.9)	317	55
Overweight (25–29.9)	126	22
Obesity (BMI > 30)	88	16

**Table 2:** Eat-26 frequency distribution.

Statement	‘0’ (%)	‘1’ (%)	‘2’ (%)	‘3’ (%)
The thought of being overweight scares me	75	12	4	9
I avoid eating when I am hungry	92	5	2	1
I find myself overly preoccupied with food-related issues	71	15	8	6
I have experimented with vomiting while feeling unable to stop	84	11	3	2
I cut my food into small pieces	88	7	4	2
I am aware of the calories in the food I eat	73	12	8	7
I specifically avoid eating carbohydrates-rich foods (bread, rice, potatoes, etc.)	86	9	4	2
I feel that others would prefer that I eat more	84	9	2	4
I vomit after meals	97	1	1	1
I feel very guilty after eating	86	8	4	2
I am preoccupied with the aspiration to be thinner	73	12	6	9
I think about burning calories while exercising	67	14	9	10
Others think that I am thin	53	14	18	15
I am disturbed by the thought of fat on my body	71	12	8	8
It takes me longer than others to finish meals	78	8	7	7
I avoid eating sugary foods	81	9	6	2
I eat diet foods	84	10	4	2
I feel that food controls my life	79	9	8	5
I demonstrate self-control regarding food	52	19	20	9
I feel pressured by others to eat	89	5	3	3
I spend too much time and thought on food	77	11	6	6
I feel uncomfortable after eating sweets	71	13	9	7
I am involved in diet-related matters	76	12	5	7
I prefer my stomach to be empty	86	6	5	3
I [do not] enjoy trying new, high-calorie foods *	32	35	21	12
I have the urge to vomit after meals	95	3	1	1

* Reversed scale. Distribution is presented after the scale was reversed.

**Table 3:** Relationships between study variables and symptoms of disordered eating.

Variable	Groups	Mean ± SD *	F/t/r	*p*
Gender	Male	8.64 ± 2.46	t_(578)_ = 2.21	0.014
	Female	10.47 ± 3.09		
In relationship	Yes	9.71 ± 2.13	t_(578)_ = 0.72	0.474
	No	10.24 ± 3.75		
Have children	Yes	8.58 ± 2.60	t_(578)_ = 3.27	0.001
	No	10.28 ± 3.60		
Religion	Jewish	9.99 ± 2.61	t_(578)_ = 0.12	0.904
	Not Jewish	9.88 ± 3.29		
Follow content related to food and eating	Yes	10.68 ± 3.04	t_(514)_ = 2.81	0.003
	No	8.63 ± 1.73		
BMI	Underweight	11.12 ± 3.07	F_(572)_ = 1.10	0.347
	Normal weight	9.39 ± 2.31		
	Overweight	10.81 ± 3.03		
	Obesity	10.39 ± 2.52		
Age			r = −0.08	0.069
Body satisfaction			r = −0.23	<0.001
Time spent on social media			r = 0.15	<0.001

* SD = Standard deviation.

## Data Availability

The original contributions presented in the study are included in the article; further inquiries can be directed to the corresponding author.
